# Extracellular Vesicles in Neuroinflammation

**DOI:** 10.3389/fcell.2020.623039

**Published:** 2021-01-21

**Authors:** Giulia Marostica, Stefano Gelibter, Maira Gironi, Annamaria Nigro, Roberto Furlan

**Affiliations:** Division of Neuroscience, Institute of Experimental Neurology, San Raffaele Scientific Institute, Milan, Italy

**Keywords:** extracellular vesicles, neuroinflammation, biomarker, multiple sclerosis, therapeutic target

## Abstract

Extracellular vesicles (EVs) are a heterogenous group of membrane-bound particles that play a pivotal role in cell–cell communication, not only participating in many physiological processes, but also contributing to the pathogenesis of several diseases. The term EVs defines many and different vesicles based on their biogenesis and release pathway, including exosomes, microvesicles (MVs), and apoptotic bodies. However, their classification, biological function as well as protocols for isolation and detection are still under investigation. Recent evidences suggest the existence of novel subpopulations of EVs, increasing the degree of heterogeneity between EV types and subtypes. EVs have been shown to have roles in the CNS as biomarkers and vehicles of drugs and other therapeutic molecules. They are known to cross the blood brain barrier, allowing CNS EVs to be detectable in peripheral fluids, and their cargo may give information on parental cells and the pathological process they are involved in. In this review, we summarize the knowledge on the function of EVs in the pathogenesis of multiple sclerosis (MS) and discuss recent evidences for their potential applications as diagnostic biomarkers and therapeutic targets.

## Introduction

Extracellular vesicles (EVs) are nano-sized vesicles released into different body fluids, heterogeneous in terms of origin, activities, compositions. Originally considered an *in vitro* artifact or useless cellular debris (Chargaff and West, 1946), they became an attractive object of research when they were shown to be an evolutionary conserved way of intercellular communication (Camussi et al., 2010; Tkach and Théry, 2016). Their precious cargo made of proteins, lipids, nucleic acids provide information of the cells and microenvironment where they are originally released.

Despite a huge advance in the knowledge of neuroinflammatory diseases, a biomarker tracking every pathogenetic step is still missing.

One of the most conceivable reason is the “hard-to-access” stage where neuroinflammation occurs. Brain and spinal cord are protected by the blood brain barrier (BBB), rather sequestered from the rest of the body and hard to be sampled.

Unsurprisingly, EVs produced by microglia, astrocyte, and other neural cells providing a snapshot of their original environment, have become the focus of thousands of studies investigating neuroinflammation, particularly in multiple sclerosis (MS).

Although recent studies have brought many advances in EV knowledge, different points still remain to be addressed. In this review we try to bridge conflicting data concerning classification, isolation, and detection techniques. Moreover, we deeply dissect EVs' role either as epiphenomenon of neuroinflammation (marker of disease) or direct culprits involved in pathogenetic mechanisms (contributory cause of disease).

## Extracellular Vesicles

### Detection and Isolation

Current approaches to identify and distinguish discrete populations of EVs are based on size, density, subcellular origin, and molecular composition (Raposo and Stoorvogel, 2013; Colombo et al., 2014; van Niel et al., 2018). Because of their overlapping physicochemical properties, classification of EVs is challenging and has produced a number of controversial results, stumbling blocks for their isolation and detection, lack of guidelines regarding their nomenclature are mainly responsible for this inconsistency. Moreover, increasing evidences highlight the existence of various subpopulations even within EV populations, suggesting that EVs are more complex entities than previously recognized and that they can be further classified into various subtypes (Lässer et al., 2018; Théry et al., 2018).

In recent years, technical advancements have led to the development of optimized methods for EV isolation and purification in attempt to elucidate the heterogeneous nature of secreted EVs and give a more comprehensive understanding on the properties of EV subtypes (Crescitelli et al., 2013; Lunavat et al., 2015; Kibria et al., 2016; Kowal et al., 2016; Willms et al., 2018). Several techniques of EV purification have been described including differential centrifugation, density gradient, ultrafiltration, polymer-based precipitation, immunoaffinity, and size exclusion chromatography, resulting in variable yield and purity of isolated EVs. Differential centrifugation remains by far the standard and the most widely used method to isolate EVs from biological fluids and media (Théry et al., 2006; Gardiner et al., 2016). This approach is often combined with density gradient flotation to efficiently eliminate copurified non-EV material or EV fragments which arise during differential centrifugation (Karimi et al., 2018; Onódi et al., 2018; Théry et al., 2018; Lee et al., 2019). In a recent work, Jeppesen et al. (2019) redefined EV composition and differential sorting of protein, RNA, and DNA between EV and non-vesicular extracellular particles. They clarified the composition of exosomes, excluding several highly abundant cytosolic proteins (like GAPDH, ENO1, and PARK7/DJ1) as well as the proteins involved in the miRNA machinery that were thought to be incorporated. Several groups have performed subfractionation of EV preparations and identified vesicle subclasses in given EV samples based on RNA composition (Crescitelli et al., 2013; Lunavat et al., 2015) or protein profiling data (Kibria et al., 2016; Kowal et al., 2016; Willms et al., 2018). Addressing the EV heterogeneity, the Lötvall's group most recently provided a detailed categorization of tumor tissue-derived EV subpopulations, separated by size and density, which showed different RNA composition and protein profile (Crescitelli et al., 2020). Interestingly, both shared and unique protein markers of EV subpopulations were found. Recently, by using asymmetric-flow field-flow fractionation, Zhang Y. et al. (2018) identified a new class of nanoparticles of 20–50 nm in diameter, named exomeres, which lack an external membrane.

A standardized approach for EVs biochemical and physical characterization is still challenging due to this huge heterogeneity concerning origin, cargo and function. Up to now, no single technology has been reported to be highly efficient to fully cover the wide spectrum of EV properties and the use of combined complementary approaches to analyze EVs is recommended. EV analytical and detection techniques are based on multiple parameters, such as size and morphology, refractive index, or presence of a certain EV-specific marker. The most popular approaches for vesicle size distribution and concentration measurements include nanoparticle tracking analysis (NTA), dynamic light scattering (DLS), and tunable resistive pulse sensing (TRPS). Flow Cytometry is also a widely use method for characterization and quantification of EVs, and even more, the high-resolution flow cytometry is reported to be a proficient tool in EV analysis, providing increased sensitivity for the detection of smaller EVs (<100 nm). In addition, non-optical methods such as immunoaffinity-based EV techniques and western blot assays have also been employed for analysis of EVs. The physical and morphological characterization of EVs is mostly performed by direct imaging with atomic force microscopy (AFM), transmission electron microscopy (TEM) and, more recently, cryo-TEM. Moreover, novel integrated lab-on-a-chip-type microfluidic devices for the detection and analysis of EVs have been developed (Coumans et al., 2017; Szatanek et al., 2017; Chiriacò et al., 2018; van der Pol et al., 2018).

Notably, the International Society for Extracellular Vesicles (ISEV) has recently suggested the minimal information required for EV research, with detailed guidelines for how to best isolate and characterize the different types of EVs (Théry et al., 2018). Combined EV isolation methods as well as improved techniques for accurate characterization are strongly recommended, toward a review of their classification, composition and biological roles. An overview of different EV isolation and detection methods is reported in Table 1.

**Table 1:** Isolation and detection methods of different EV subtypes.

**EV subtypes**	**Size range**	**Biogenesis**	**Isolation**	**Detection**
**Exosomes (Exo)** (Théry et al., 2006, 2018; Raposo and Stoorvogel, 2013; Yáñez et al., 2015; Kowal et al., 2016; Willms et al., 2018; Jeppesen et al., 2019)	30–100 nm	Release by exocytosis of multivesicular bodies (MVBs)	Differential centrifugation and density gradients (100,000–200,000 × g), immunoprecipitation, commercial kit, size exclusion chromatography	TEM, AFM, NTA, TRPS, DLS, WB, flow cytometry (bead coupled), ELISA
**Microvesicles (MVs)** (Lötvall et al., 2014; Cocucci and Meldolesi, 2015; Kowal et al., 2016; Szatanek et al., 2017; Chiriacò et al., 2018; Crescitelli et al., 2020)	100–1,000 nm	Direct budding of the cell membrane	Differential centrifugation (10,000–20,000 × g) Density gradients	TEM, AFM, WB, flow cytometry (for vesicles >300 nm)
**Apoptotic bodies** (Wickman et al., 2012; Atkin-Smith et al., 2015; Xu et al., 2019)	500–5 μm	Outward blebbing and fragmentation of the cell membrane	Centrifugation, filtration	TEM, IF, flow cytometry

*TEM, transmission electron microscopy; AFM, atomic force microscope; NTA, nanoparticle tracking analysis; TRPS, tunable resistive pulse sensing; DLS, dynamic light scattering; IF, immunofluorescence microscopy; ELISA, enzyme-linked immunosorbent assay; WB, western blotting*.

### Classification

**Figure 1:**
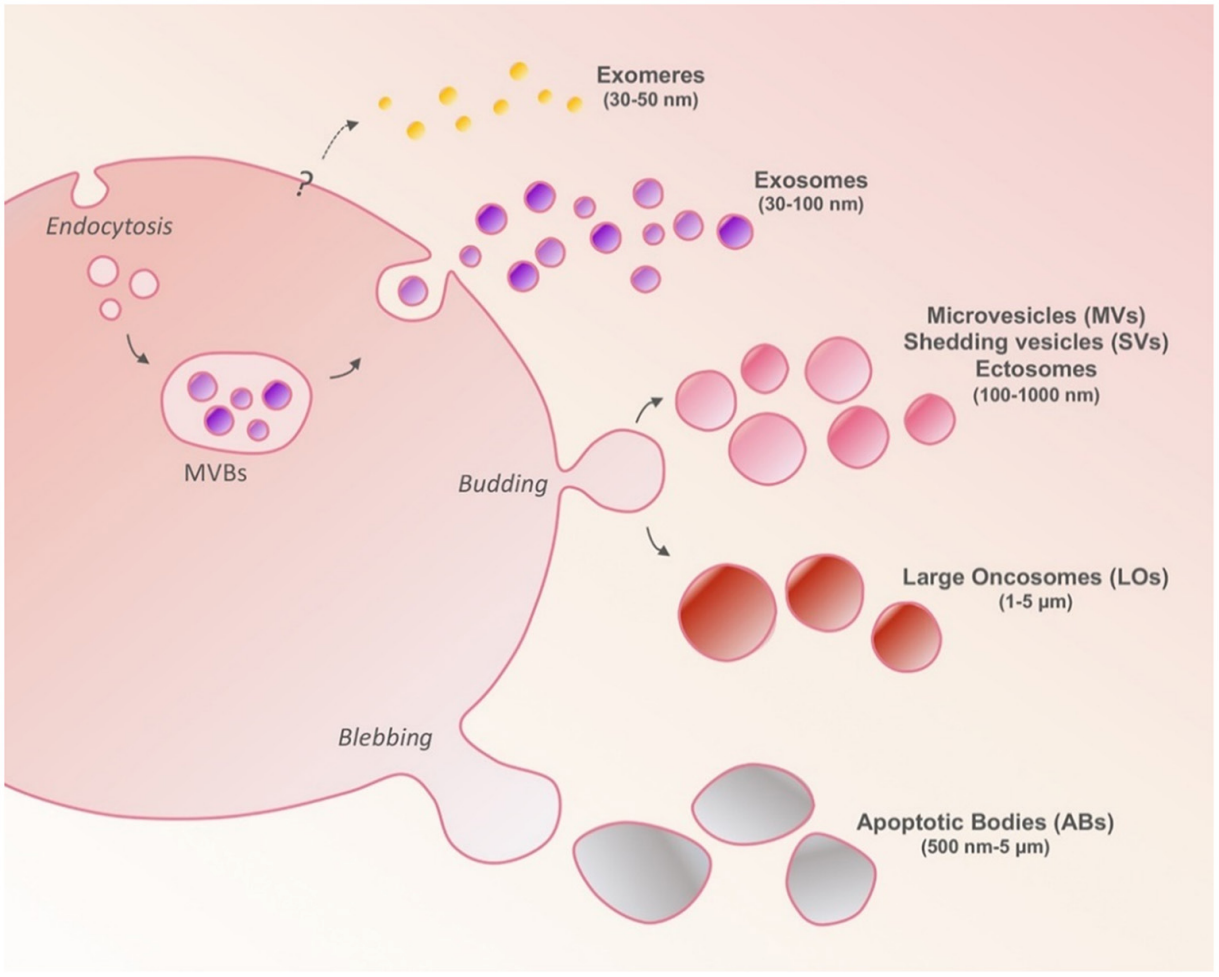
Cells release heterogeneous populations of EVs different in sizes and secretion pathways. Exosomes are generated intracellularly from multivesicular bodies (MVBs). Microvesicles (MVs) are larger than exosomes and arise as a result of outward budding and fission of the plasma membrane. Large oncosomes (LOs) are derived from the shedding of non-apoptotic blebs unique to cancer cells. Apoptotic bodies are released upon cell fragmentation during apoptotic cell death. Exomeres have been recently suggested to be non-membranous nanoparticles with size smaller than 50 nm; their biological role remains unknown.

A conventional EV classification is based on their biogenesis and biophysical characteristics and distinguishes EVs into three major populations: exosomes, microvesicles (MVs), and apoptotic bodies. However, this classification should be considered with caution in line with recent evidences that report the presence of other subsets of EVs and add a new layer of complexity that need to be addressed in EV field (Figure 1).

Exosomes are small extracellular nano-size vesicles, typically 30–100 nm in diameter that are formed as intraluminal vesicles within endosomal multivesicular bodies (MVBs), and are released from cells upon the fusion of MVBs with the plasma membrane (Raposo and Stoorvogel, 2013). MVBs can also fuse with lysosomes to degrade the content. The outward budding of exosomal membranes can be regulated by the endosomal sorting complex required for transport (ESCRT) proteins or by neutral sphingomyelinase and ceramide in an ESCRT-independent manner (Stuffers et al., 2009). Due to their endocytic origin, exosomes can be identified by specific markers such as proteins involved in MVB biogenesis (ALIX and TSG101), membrane transport and fusion proteins (GTPases, Annexins, and flotillin) and tetraspanin proteins (CD9, CD63, CD81) (Mathivanan and Simpson, 2009). Exosomal membranes are enriched in elements of lipid rafts (e.g., GM1 gangliosides) and lipid components including cholesterol, ceramide, and sphingomyelin (Yáñez et al., 2015).

MVs, also known as ectosomes or shedding vesicles (SVs), are larger than exosomes ranging in size typically from 100 to 1,000 nm in diameter (Cocucci and Meldolesi, 2015). They originate directly by the outward budding of the plasma membrane and are subsequently released into the extracellular space after a selective incorporation of proteins, nucleic acids and lipids (Tricarico et al., 2016). The outer leaflet of the MV membrane is enriched with phosphatidylserine and sphingomyelin but also cholesterol and transmembrane proteins, which enable MV shedding through charges in plasma membrane shape (Muralidharan-Chari et al., 2010). Common markers used for identifying exosomes, such as CD63 and CD81 tetraspanins or components of the endosomal complex, such as TSG101 and ALIX, can also be found in MVs. However, CD40 ligand, ADP-ribosylation factor 6 (ARF6), HSP90, gp96 and different protein associated to lipid rafts, such as integrins and flotillins, are reported as MV markers (Muralidharan-Chari et al., 2009; Kim et al., 2012; Lötvall et al., 2014). Recently, Annexin A1 has been proposed as a novel and specific marker for MVs (Jeppesen et al., 2019).

Apoptotic bodies are 500 nm−5 μm in diameter and are released into the extracellular environment during the cell apoptotic process (Muhsin-Sharafaldine and McLellan, 2018; Xu et al., 2019). They contain fragmented subcellular organelles for degradation, and they can be characterized by cellular organelles and DNA (Caruso and Poon, 2018).

Due to the substantial size overlap among these membrane vesicles, confusion on their nomenclature has spread throughout the literature, limiting a consensus on understanding the specific biological functions of different categories of vesicles. Along with the definition of a uniformly accepted EVs nomenclature, further studies specifically addressing EVs subtypes are needed in order to better standardize results obtained in the field. For clarity, in this review, we will specify/discriminate exosomes and MVs exclusively in studies concerning MS allowing for this distinction and use the term EVs when both types of vesicles are investigated.

## EVs in CNS and Neuroinflammation

### EVs in Neurological Diseases

The CNS is a complex organ comprising several cell types, each exerting a unique function. Neurons, astrocytes (Thompson et al., 2016), oligodendrocytes (Frühbeis et al., 2012), and microglia (Verderio et al., 2012) have all been reported to release EVs *in vitro* and *in vivo*, contributing in physiological and pathological conditions in the brain. EVs are involved in transporting signals between neighboring cells within the CNS (Basso and Bonetto, 2016; van der Vos et al., 2016), and are implicated in other processes, including development, synaptic neurotransmission, neurodegeneration, and tumor progression.

Thanks to their biological characteristics, EVs spread around the CNS but can easily cross the BBB and diffuse into peripheral blood and reach other organs. Several mechanisms have been proposed, however it seems that exosomes can cross the BBB by moving from cell to cell *via* MVB compartment and endocytosis (Record et al., 2011). More recently, exosomes released by a human glioma transplanted in mouse brain were detected in peripheral blood, crossing an intact BBB (García-Romero et al., 2017).

One notable example for the study of EVs spreading in the periphery, comes from the study of a mouse model of CNS injury. Exosomes isolated from peripheral blood of damaged brain mice where intravenously injected into naïve mice, causing an immune cell activation in the liver of recipient mice (Couch et al., 2017). This is the first study which strongly indicates an immune activation of cells in CNS disease mediated by exosomes.

According to the possibility for the EVs to cross both undamaged and disrupted BBB, EVs could be detected in different biological fluids and represent a new class of biomarkers (Verderio et al., 2012; Galazka et al., 2017) and be also implicated in some stage of MS development and pathogenesis (Dolcetti et al., 2020).

### MS Pathogenesis

MS is a chronic inflammatory autoimmune demyelinating disease of the CNS (Harbo et al., 2013). It affects worldwide more than 2 million people, being the most common immune mediated CNS disease. MS has a typical onset between the ages of 20–40 years, women outnumbering men 3:1, being for both sexes the leading cause of non-traumatic disability in young adults (Harbo et al., 2013).

Clinically heterogeneous, MS can be classified into different disease phenotypes. These include relapsing remitting MS (RRMS), secondary progressive MS (SPMS), primary progressive MS (PPMS), and clinically isolated syndrome (CIS) (Lublin et al., 2014). The most common form is RRMS, characterized by acute episodes of neurological deficit (relapses) followed by partial or complete clinical remission (Weiner, 2008). On the opposite, the progressive forms of MS are characterized by insidious disability accrual over time that can start ab initio (PPMS) or after a RR phase (SPMS). CIS is defined as the first clinical manifestation of a diseases that shows features of demyelination typical of MS, not fulfilling yet MS diagnostic criteria (Lublin et al., 2014). In addition, radiologically isolated syndrome (RIS) is characterized MRI incidental findings of CNS lesions suggestive of demyelination (Lebrun-Frenay et al., 2020). Inflammation, demyelination, gliosis and axonal degeneration are major histopathological hallmarks, responsible for different and unpredictable clinical courses (Ransohoff et al., 2015). Despite huge advances in its neurobiology, neuroimaging, neuropathology, MS remains a disease of unknown etiology. Epidemiological data suggest an etiological role for both genetic and environmental factors. Biological findings point to neuroinflammation and neurodegeneration as intertwined mechanisms involved in MS pathogenesis (Ransohoff et al., 2015). Crucial players of neuroinflammation are dysfunctional cells of both innate and adaptive immune system. Monocytes and peripherally activated T and B cells transmigrate from the blood to the CNS where they start myelin and axonal disruption (Lassmann, 2019). From peripheral activation to neural destruction, almost all pathogenetic steps have been largely studied and various mechanisms have been elucidated. A defective functioning of FoxP3-CD4^+^ CD25^high^ regulatory T cells exosomes is recognized as contributing to an insufficient suppression, leading to a possible mechanism of activation of autoreactive T cells in periphery (Azimi et al., 2018). Upon activation these cells transmigrate across BBB by a multistep process where endothelial adhesion molecules and cognate ligand on leukocytes are involved (Brandstadter and Katz Sand, 2017).

The most convincing evidence of how crucial this step is, is provided by the effectiveness of natalizumab. This disease modifying drug interferes with an adhesion molecule (integrin-α4) leading to a robust decrease in clinical and radiological disease activity in most of the treated RRMS patients (Havrdova et al., 2009). Once in the CNS, these cells undergo further activation and together with microglia orchestrate myelin and axonal destruction (Lassmann, 2019). A proinflammatory environment caused by cytokines and chemokines, proteolytic enzymes and neurotoxic products leads to neuroinflammation, demyelination and neurodegeneration occurring through CNS white and gray matter.

Since a long time, neurodegeneration has been recognized as crucial pathogenetic mechanism in MS, already present in the early stages of disease. Among others, oxidative stress, iron accumulation, mitochondrial injury, ion channel dysfunction, glutamate excitossicity are widely found in MS neurodegeneration (Lassmann, 2019).

Unfortunately, when, how and to what extent these phenomena get into action is still elusive.

### EVs Role in MS

In 2017 (Mustapic et al., 2017) L1 cell adhesion molecule (L1CAM) was appointed as a promising marker for EVs derived from CNS region. The possibility of identifying EVs of neural origin represent a precious messenger of tissue releasing them. Deciphering their origin and biogenesis can provide new insight into MS pathogenetic mechanisms still partially understood. Conflicting results about EVs role in MS can be ascribed to different methods of studies but also to a Janus role that EVs play in MS. Depending on cell of origin, microenvironment, status of disease, EVs can be detrimental or protective.

Studies *in vitro* have shown that endothelium-derived EVs are involved in the activation of CD4^+^ and CD8^+^ T lymphocytes (Wheway et al., 2014) and can cause their infiltration in the CNS.

One of the most accredited mechanisms postulate that EVs carrying metalloproteinases have a key role in BBB disruption (Sáenz-Cuesta et al., 2014). Again, recent studies demonstrated that EVs derived from brain microvascular endothelial cells can provide a bridge protein (claudin-5) between leukocytes and endothelial cells inducing transendothelial migration of the former (Paul et al., 2016).

An indirect confirmation of detrimental role of EVs in MS pathogenesis comes from experimental autoimmune encephalomyelitis (EAE), mouse model of MS. A-SMase KO mice, genetically impaired in MVs production, are protected from EAE (Verderio et al., 2012).

By contrast, several studies suggest a repairing activity of EVs. Indeed, microglia exposed to IL4 or mesenchymal stem cell (MSC) can release EVs promoting oligodendrocyte precursors cells (OPC) migration and/or differentiation. Lipids embedded in EV surface have being indicated as crucial mediators of this myelin repairing activity (Lombardi et al., 2019). Using EVs as therapeutic vector to mediate a protective and repairing activity during the disease will be further explored in the review.

Again, one of the first studies focusing on EVs (Scolding et al., 1989) reported that oligodendrocytes can recover from injury by “membrane-attack complex-enriched vesicles” released from the surface of viable cells.

A protective function of EVs was further studied in MS during pregnancy. It is known that MS female patients experience less severe symptoms and less frequent relapse during pregnancy (Airas and Kaaja, 2012). Studies in the animal model showed that serum derived EVs from mice in late pregnancy were more numerous than those isolated from virgin mice (Gatson et al., 2011). Moreover, T cells co-cultured with exosomes from pregnant mice induced stronger suppression of T-cell proliferation compared to exosomes obtained from virgin animals (Gatson et al., 2011). A further study by the same group showed a reduction in IFN- gamma production and decreased expression of Tbet (Th1 transcription factor) in T cells exposed to pregnancy-derived exosomes. In addition, the authors demonstrated, in EAE, the positive effect of pregnancy-derived exosomes on oligodendrocyte precursor cells migration to lesion areas and subsequent maturation (Williams et al., 2013). Accordingly, authors conclude that EVs are among crucial agents responsible for the immune modulation in pregnant EAE mice.

Besides “conventional” immune cells, myeloid cells, such as infiltrating macrophages and resident microglia, have been indicted in MS pathogenesis. Like EVs, their role is dual, acting either as harmful or as protective factor. Indeed, activated myeloid cells release EVs detectable in CSF of MS patients and animal models (Carandini et al., 2015; Nigro et al., 2016). For example, pro-inflammatory cytokines such as IL1β, IFNγ, TNFα, and caspase 1 can be loaded inside EVs and boost an inflammatory microenvironment. As previously mentioned, EVs contain metalloproteinases responsible for degradation of the extracellular matrix and tight junctions, facilitating leukocytes infiltration (Sáenz-Cuesta et al., 2014; Zappulli et al., 2016). Consequently, injection of microglia-derived EVs into EAE mice led to a recruitment of inflammatory T-cells and exacerbated the disease (Verderio et al., 2012; Sáenz-Cuesta et al., 2014).

Another source of EVs is represented by those released by astrocytes. In response to neuronal injury, astrocytes become activated and release EVs containing IL-1β, which exacerbates tissue deterioration, fostering a vicious circle (Prada et al., 2018).

## EVs in MS Clinical Practice

### EVs as Biomarkers

Despite of a large research effort, several biomarkers have been proposed for MS, but their clinical relevance remains uncertain (Selmaj et al., 2017; Ziemssen et al., 2019). Complex diseases require biomarkers that allow to read into their complexity. In this direction, EVs seem to be good candidates. They are known to cross the BBB, allowing CNS EVs to be detectable in peripheral fluids (Verderio et al., 2012; Galazka et al., 2017; Shi et al., 2019) and their unique content could give information about the origin cells and on the pathological process they are involved in. Considering this, interest in EVs as a potential biomarker in neuroinflammatory diseases is constantly increasing. The number of original researches dealing with EVs in MS has more than quadrupled in the last 5 years, compared to the same previous period.

We identified 27 original researches on the PubMed Database relating to EVs as biomarker in MS (see Table 2) published from 2011 to early 2020. Sixteen of these works analyzed plasma or serum-derived EVs, seven from CSF, six from the supernatant of cell cultures or PBMC, one from urine and one from tears. In nine papers the miRNA contained in the EVs was investigated. Seven papers were focused on differences in concentration of EVs with specific surface markers, while three studies investigated concentration or production of the total EVs population. Five publications analyzed EVs protein content, whereas two investigated lipid cargo. Finally, three papers were focused on functional assays.

**Table 2:** EVs as biomarkers in MS.

**References**	**Patients**	**EV**	**Isolation method**	**Quantification method**	**Source**	**Cargo/measures**	**Results**	**Biomarker**
Azimi et al. (2019)	RRMS, HC	Exo	Total exosome isolation kit (Invitrogen)	Elisa	T cell cultures	miRNA	miR-326 is upregulated in RRMS vs. HC.	Diagnostic
Bhargava et al. (2019)	RRMS, HC	Exo	Exoquick (System Biosciences)	NTA—nanosight	Serum	Concentration protein	TLR3 reduction, TLR4 increase in RRMS vs. HC.	Diagnostic
Ebrahimkhani et al. (2020)	RRMS	Exo	Size exclusion chromatography (qEV Izon)	NTA—nanosight	Serum	miRNA	miRNA differential expression between active vs. non-active RRMS. miRNA differential expression between FTY responders vs. non-responders.	Disease activity, response to treatment
Pieragostino et al. (2019)	MS, HC	EVs	FACS	FACS, Dynamic light scattering	CSF, Tears	Proteomics	Microglial and neuronal EVs detectable in CSF and tears. Protein cargo is different between MS and HC. Protein cargo overlap (70%) between tears and CSF EV in MS.	Diagnostic
Amoruso et al. (2018)	RRMS, HC	MV	Differential centrifugation	Fluorescence	Monocytes	Concentration	Increased MVs concentration in RRMS vs. HC, reduced by FTY	Diagnostic, treatment effect
Azimi et al. (2018)	RRMS, HC	Exo	Total exosome isolation kit (Invitrogen)	Elisa	Treg cultures	Functional assay	Treg-derived MS exo are less effective in suppressing conventional T cell proliferation and in inducing T cell apoptosis.	Diagnostic
Geraci et al. (2018)	RRMS, OND	EV	Differential centrifugation	FACS, NTA—nanosight	CSF	Concentration Markers	No differences in concentration and Ib4 positivity in MS vs. OND. Increased concentration, Ib4 positivity and CD19^+^/CD200^+^ in active vs. stable MS. CCR3^+^ CCR5^+^ CD4^+^/CCR3^+^, CD4^+^/CCR5^+^ CC3^+^/CCR5^+^ are increased in MS with MRI activity.	Disease activity
Kimura et al. (2018)	RRMS, PMS, HC	Exo	Differential centrifugation	NTA—nanosight	Plasma	Functional assay, miRNA	let-7i, miR-19b, miR-25, miR-92a are upregulated in MS. No differences between disease subtype. MS exo decrease Treg cell frequency, through let-7i.	Diagnostic
Manna et al. (2018)	RRMS	Exo	Exoquick (System Biosciences)	Dynamic light scattering	Serum	miRNA	miRNA differential expression between IFNB-treated vs. naive RRMS. miRNA differential between IFN responders vs. non-responders	Treatment effect, Response to treatment
Pieragostino et al. (2018)	MS, c-OND, p-OND	Exo	FACS	FACS, Dynamic light scattering	CSF	Lipids	Increased Exo concentration in MS vs. to p- and c-OND. Concentration correlates with acid sphingomyelinase activity. Exo deliver active acid sphingomyelinase cargo. Exosomal acid sphingomyelinase activity correlates with EDSS.	Diagnostic, Disability
Sáenz-Cuesta et al. (2018)	RRMS, HC	EVs	Differential centrifugation	NTA—nanosight	Serum	Concentration miRNA Functional assays	FTY increases EVs concentration. FTY changes EVs miRNA expression. FTY reduces the ability of EVs to inhibit lymphocyte activation.	Treatment effect
Blonda et al. (2017)	RRMS, HC	MV	Differential centrifugation	Fluorescence	Monocytes	Production	Increased MVs concentration in MS. IFNB, TFM, and FTY reduce monocyte MVs production.	Disease, Treatment effect
Dalla Costa et al. (2018)	RRMS, HC	MV	Differential centrifugation	FACS	Monocytes	Concentration	Increased MV concentration in RRMS vs. HC. FTY reduces concentration, NTZ increases it. No differences for IFN and GA.	Diagnostic, Treatment effect
Ebrahimkhani et al. (2017)	MS (RR, PMS), HC	Exo	Size exclusion chromatography (qEV Izon)	NTA—nanosight	Serum	miRNA	miRNA are dysregulated in MS. Differential expression in disease subtype. Combination of 3 or more miRNAs predicts the clinical form.	Disease, Disease subtype
Galazka et al. (2017)	MS (RR, PMS), HC	Exo	Exoquick kit (System Biosciences)	NTA—nanosight	Serum, CSF	Concentration protein, Functional assay	MOG is increased in RRMS and SPMS. MOG correlates with MRI activity. MS exo induces proliferation of MOG-TCR transgenic T cells.	Diagnostic, Disease activity
Niwald et al. (2017)	RRMS, HC	Exo	Total exosome isolation kit (Life Technologies)	None	Serum	miRNA	miR155, miR-301a decrease and miR-326 increase in MS. miR-301a and miR155 are higher in recently active RRMS.	Diagnostic, Disease activity
Selmaj et al. (2017)	RRMS, HC	Exo	ExoQuick kit (System Biosciences)	NTA—nanosight	Serum, PBMC	Concentration miRNA	4 miRNA are differentially expressed among HC, aRRMS, naRRMS. Negative correlation with MRI activity and clinical activity. These miRNA are significantly less concentrated in RRMS PMBC exo.	Diagnostic, disease activity
Welton et al. (2017)	RRMS vs. IIH	Exo	Exo-spin (Cell GS)	NTA—nanosight	CSF	Concentration proteomics	Higher concentration and p/p ratio in RRMS. 50 proteins specifically enriched in RRMS exo vs. RRMS CSF.	Diagnostic,
Lee et al. (2019)	MS, NMO, LETM	Exo	Differential centrifugation	FACS, NTA—nanosight	CSF	Proteomics	MS and NMO have a different exosomal protein content.	Diagnosis
Moyano et al. (2016)	RRMS, HC	Exo, MV	Differential centrifugation	NTA—nanosight	Plasma	Concentration size lipids	C16:0 sulfatide is more expressed in RRMS vs. HC. Negative correlation with EDSS.	Diagnostic, Disability
Zinger et al. (2016)	RRMS, HC	EV	Differential centrifugation	FACS	Plasma	Concentration markers	Total EV and CD105^+^ MPs are increased while CD19^+^ EV are reduced in untreated RRMS vs. HC. FTY restores their levels comparable to HC.	Diagnostic, Treatment effect
Alexander et al. (2015)	MS (RR, SP), HC	Exo	Differential centrifugation	FACS	Plasma	Concentration markers	Exo from different sources are differently modulated in RR and SPMS. Correlation with MRI measures.	Diagnostic
Giovannelli et al. (2015)	RRMS, HC	Exo	Exosome-specific extraction kit (Norgen)	None	Plasma, urine	miRNA	JCV miRNA are more represented in exo of JCV + RRMS (NTZ) and HC.	Treatment side effects
Marcos-Ramiro et al. (2014)	MS (CIS, RR, PMS), HC	EV	Differential centrifugation	FACS	Plasma	Concentration markers	CD62^+^ and CD31^+^ are increased in all MS subtypes vs. HC	Diagnostic
Sáenz-Cuesta et al. (2014)	RRMS, SPMS, HC	MV	Differential centrifugation	FACS	Plasma	Concentration markers	CD61^+^, CD45^+^, CD14^+^ MP are increased in RRMS vs. SPMS and HC. NTZ and IFNB treatment increase their level.	Diagnostic, treatment effect
Verderio et al. (2012)	MS, NMO, OIND, ONIND, HC	MV	FACS	FACS	CSF	Concentration of myeloid MVs	Myeloid MVs are increased in active RRMS compared to stable RRMS and in CIS compared to HC. MVs concentration correlates with MRI activity.	Diagnostic disease activity
Nordberg et al. (2011)	MS	EV	FACS	FACS	Plasma	Concentration markers	CD31^+^ and CD54^+^ MPs are reduced by IFNB. Correlation with a reduction of MRI activity.	Response to treatment

*MS, multiple sclerosis; RR, relapsing remitting; PMS, progressive multiple sclerosis; SP, secondary progressive; HC, healthy controls; OND, other neurological disorders; IIH, idiopathic intracranial hypertension; NMO, neuromyelitis optica; LETM, longitudinal extensive transverse myelitis; CIS, clinically isolated syndrome; ONIND, other non-inflammatory neurological disorders; Exo, exosomes; MV, microvesicles; NTA, Nanoparticle tracking analysis; PBMC, peripheral blood mononuclear cell; CSF, cerebrospinal fluid; miRNA, microRNA; FTY, fingolimod; IFNB, interferon-beta; TFM, teriflunomide; NTZ, natalizumab; EDSS, expanded disability status scale; MOG, myelin oligodendrocyte glycoprotein*.

To the date, none of the published works reached the ambitious target of a possible use of EVs in MS clinical practice. Papers focusing on CSF-EVs demonstrated good correlations with MS disease activity or disability (Verderio et al., 2012; Geraci et al., 2018; Pieragostino et al., 2018), in some cases supporting also a role in the diagnostic process (Verderio et al., 2012). Nevertheless, since CSF collection requires an invasive procedure, CSF-EVs don't meet the need of an easily-collectable biomarker (Ziemssen et al., 2019), preventing their use in monitoring disease over time. Serum/plasma-derived EV miRNAs were found to be possible markers of disease activity (Niwald et al., 2017; Selmaj et al., 2017; Ebrahimkhani et al., 2020), together with serum exosomal MOG protein (Galazka et al., 2017). EV miRNAs were also proposed as potential biomarkers of treatment response (Manna et al., 2018; Ebrahimkhani et al., 2020), as well as platelet-derived EVs (Nordberg et al., 2011), whereas lipid content correlates with disability (Moyano et al., 2016). Nonetheless, taking into account the complexity and cost of EV isolation and characterization, these findings do not provide yet an added value to clinical and MRI management of MS patients (Ziemssen et al., 2019). Interestingly, Ebrahimkhani et al. (2017) proposed miRNA signatures able to distinguish RRMS from PMS, addressing a dramatically open issue of this disease.

However, a common limit of all the studies about blood EVs is that they do not specifically address CNS-derived EVs. Recently, Goetzl et al. (2015) published a method to enrich plasma exosomes for neuronal origin. This method was then replicated and applied also for astrocyte-derived (Goetzl et al., 2016, 2018) and oligodendrocyte-derived exosomes (Dutta et al., 2018). Since CNS-derived exosomes represent a very small percentage of the total plasma/serum exosome population (Hornung et al., 2020), the employment of CNS-derived exosomes enriching techniques might lead to peripherally collectable CNS biomarkers in MS, as demonstrated in other neurological diseases (e.g., Alzheimer disease, brain traumatic injury) (Goetzl et al., 2015, 2019).

The wide variability in isolation methods and analysis (see Table 2) makes an organic interpretation of the published works at least challenging. According to comparison studies, different isolation methods have different performances concerning purity of samples and co-isolation of contaminating material (Baranyai et al., 2015; Lee et al., 2019; Takov et al., 2019). In light of this, Rekker et al. (2014) observed that exosomal miRNA profile is affected by the isolation method (i.e., Ultracentrifugation vs. ExoQuick). Tang et al. (2017) demonstrated similar results. The choice of different methods among authors [not always in agreement with those suggested by ISEV (Théry et al., 2018)] might affect the reliability and consistency of the findings, both for cargo analysis and functional assays (Takov et al., 2019). Finally, as discussed above, Jeppesen et al. (2019) had recently published an important paper that strongly stresses the need for exosome composition reassessment, changing the whole EVs research scenario. In this direction, a further validation of what previously published about EVs and MS should be required.

### EVs as Therapeutic Tool

MS and its animal model EAE are the most common inflammatory demyelinating diseases caused by autoimmune-activated immune cells in the CNS (Li et al., 2017; Zhang Y. et al., 2018). It has been reported that EVs can penetrate the BBB and contribute to brain antigens spreading to the periphery (Selmaj et al., 2017). The injection of microglia-derived EVs into the CNS of EAE mice enhanced inflammation and exaggerated disease (Verderio et al., 2012). Moreover, mice with an impaired ability to secrete EVs were resistant to EAE (Verderio et al., 2012). Accordingly, it's conceivable that EVs are involved in EAE pathogenesis.

Based on the possible role of EVs in EAE and MS, some groups developed treatment strategies that employed the use of EVs as therapeutic tool to treat neuroinflammation and demyelination.

The use of MSCs has always given good outcomes in the treatment of EAE and is currently in clinical trial for MS (Karussis et al., 2010; Yamout et al., 2010; Cohen, 2013). Nevertheless, cell-based therapies are affected by the immune rejection of donor cells among main safety issues (Kim and Park, 2017). To overcome this problem, several groups are now interested in eliminating the rejection problem by administering a non-cell based treatment, namely EVs. MSCs stimulated with IFNγ produce exosomes carrying these cytokines, and their administration results in a good therapeutic effect on EAE (Riazifar et al., 2019). Again, MSCs derived from placenta were found effective in having a regenerative, immunomodulatory and protective effect. The exosomes derived from this type of cells can reduce DNA damage in oligodendroglia and increase myelination within the spinal cord of treated mice (Clark et al., 2019). Conversely, bone marrow derived MSCs exosomes, administered systemically, can decrease the immune cell infiltration and inflammation in the CNS, together with decreasing the demyelinating process (Zhang Q. et al., 2018). In the same way, another group, using bone marrow derived MSCs pre-treated with IFNγ has shown a decreased demyelination in EAE mice and manage to demonstrate that the administration of exosomes can ameliorate EAE by suppressing pathological Tcells activation and inducing Tregs action (Riazifar et al., 2019). Moreover, these exosomes seems to mediate a phenotype switch of microglia from a pro-inflammatory to a rescue phenotype (Li et al., 2019). These data provided evidence that MSCs-derived exosomes can be used as cell-free therapies for autoimmune and central nervous system diseases.

In order to elicit a specific reaction, scientists use different cell source of EVs to hit specific target cells.

Accordingly, the use of EVs derived from dendritic cells overexpressing TGF-β1 resulted in the inhibition of Th1 and Th17 differentiation and T reg cells were promoted, leading to milder EAE (Yu et al., 2013). Casella et al. designed a mouse microglial cell line releasing a large amount of engineered EVs containing the anti-inflammatory cytokine IL-4 and expressing a target eat-me signal for macrophages on the surface. EAE mice injected with these engineered EVs show a less severe course of disease (Casella et al., 2018). Through intranasal route, Zhuang et al. (2011) manage to deliver curcumin-loaded exosomes in CNS and manage to reduce neuroinflammation by targeting resident microglia. Other than microglia, oligodendrocytes and their differentiation are an interesting target in treating demyelination in EAE. Pusic et al. (2014) used exosomes from bone marrow dendritic cells to support oligodendrocytes maturation. Exosomes produced by IFNγ stimulated dendritic cells contain high levels of miR-219, a key player in oligodendrocyte precursors cells differentiation. In addition, these exosomes can increase the number of mature oligodendrocytes and help the remyelination process *in vivo*.

Compared to the crucial contribution and successful results of EVs in cancer treatment, EVs applications as therapeutic delivery vehicle in neuroinflammatory diseases are yet in their early stages (Villa et al., 2019; Hernandez-Oller et al., 2020; Li et al., 2020). Currently just one clinical trial is ongoing for the treatment of chronic postsurgical temporal bone inflammations (Clinicaltrials.gov, NCT04281901), but we are sure that in the next few years the number of clinical trials using EVs as therapeutic vector will increase.

## Conclusions

There is increasing evidence, as described in this review, supporting a pivotal role for EVs both in CNS physiology and during neuroinflammatory pathogenetic processes. Most of our knowledge on the role of EVs in the CNS comes, however, from *in vitro* studies, since technologies to follow EVs fate *in vivo* are difficult and, in any case, uncertain. The development of tools allowing the modulation of CNS EVs release and/or uptake *in vivo* in animal models would represent a ground-breaking advancement for the field. This would allow to highlight EVs role in physiological and pathological processes, *in vivo* and possibly in a cell-specific manner. Unfortunately, available genetic models lack specificity and most pathways (and therefore genes) involved in EVs release or uptake have yet to be elucidated. Specific pharmacological tools, on the other hand, are also missing.

The use of EVs as biomarkers in clinical practice seems a more realistic short-term goal. As demonstrated by many studies, EVs can mirror the biology and environment of the donor cell, providing a broader information on ongoing pathologies in neurological patients. Both their number and content, comprising different kind of signals, may provide indication of disease stage and prognosis. Access to pathologically relevant tissues is extremely difficult for neurological diseases, thus the use of EVs may be key since may be easily isolated from the blood. What we still miss here is a deeper knowledge of EVs subpopulations in the blood, to differentiate and better characterize EVs origin and their content in physiological or pathological conditions. The uncertain and evolving technological landscape for EVs examination calls for a consensus on protocols, for standardization of EVs isolation and analysis. International associations (i.e., ISEV) are trying to do this work but cannot avoid the publication of studies of heterogeneous quality.

Finally, the use of EVs as therapeutic vectors in neurological diseases is just at its beginning. There are some encouraging data and publications but many more pre-clinical studies must be performed before establishing ways of administration, nature of plausible cargos, *in vivo* fate, possible target cells, etc. The study of the therapeutic use of EVs in the cancer field is more advanced and raises hopes we might develop EVs as therapeutic vectors in clinical trials in the future also for neurological disorders.

There are still several technical challenges and even more learning needs on EVs biology, but we are convinced that current difficulties in this research field may be overcome, and that EVs may become in the next years not only clinically useful biomarkers, but also a source of information on disease pathogenesis and possibly an alternative therapy for currently untreatable diseases.

## Author Contributions

GM, SG, MG, and AN wrote the review and correct the text. RF supervised the writing of the review and correct the final form of the article. All authors contributed to the article and approved the submitted version.

## Conflict of Interest

The authors declare that the research was conducted in the absence of any commercial or financial relationships that could be construed as a potential conflict of interest.

## References

[B1] Airas L. Kaaja R. (2012). Pregnancy and multiple sclerosis. *Obstet*. Med. 5, 94–97. 10.1258/om.2012.110014 27582863 PMC4989704

[B2] Alexander J. S. Chervenak R. Weinstock-Guttman B. Tsunoda I. Ramanathan M. Martinez N.. (2015). Blood circulating microparticle species in relapsing–remitting and secondary progressive multiple sclerosis. A case–control, cross sectional study with conventional MRI and advanced iron content imaging outcomes. J. Neurol. Sci. 355, 84–89. 10.1016/j.jns.2015.05.027 26073484 PMC4550483

[B3] Amoruso A. Blonda M. D'Arrigo G. Grasso R. Di Francescantonio V. Verderio C.. (2018). Effect of fingolimod action on the release of monocyte-derived microvesicles in multiple sclerosis patients. J. Neuroimmunol. 323, 43–48. 10.1016/j.jneuroim.2018.07.008 30196832

[B4] Atkin-Smith G. K. Tixeira R. Paone S. Mathivanan S. Collins C. Liem M.. (2015). A novel mechanism of generating extracellular vesicles during apoptosis via a beads-on-a-string membrane structure. *Nat*. Commun. 6:7439. 10.1038/ncomms8439 26074490 PMC4490561

[B5] Azimi M. Ghabaee M. Moghadasi A. N. Noorbakhsh F. Izad M. (2018). Immunomodulatory function of Treg-derived exosomes is impaired in patients with relapsing-remitting multiple sclerosis. *Immunol*. Res. 66, 513–520. 10.1007/s12026-018-9008-5 29882035

[B6] Azimi M. Ghabaee M. Naser Moghadasi A. Izad M. (2019). Altered expression of miR-326 in T cell-derived exosomes of patients with relapsing-remitting multiple sclerosis. *Iran. J*. Allergy Asthma Immunol. 18, 108–113. 10.18502/ijaai.v18i1.636 30848579

[B7] Baranyai T. Herczeg K. Onódi Z. Voszka I. Módos K. Marton N.. (2015). Isolation of exosomes from blood plasma: qualitative and quantitative comparison of ultracentrifugation and size exclusion chromatography methods. PLoS ONE 10:e0145686 10.1371/journal.pone.0145686 26690353 PMC4686892

[B8] Basso M. Bonetto V. (2016). extracellular vesicles and a novel form of communication in the brain. *Front*. Neurosci. 10:127. 10.3389/fnins.2016.00127 27065789 PMC4814526

[B9] Bhargava P. Nogueras-Ortiz C. Chawla S. Bæk R. Jørgensen M. M. Kapogiannis D. (2019). Altered levels of toll-like receptors in circulating extracellular vesicles in multiple sclerosis. Cells 8:1058. 10.3390/cells8091058 31509962 PMC6769450

[B10] Blonda M. Amoruso A. Grasso R. Di Francescantonio V. Avolio C. (2017). Multiple sclerosis treatments affect monocyte-derived microvesicle production. Front. Neurol. 8:422. 10.3389/fneur.2017.00422 28878732 PMC5572278

[B11] Brandstadter R. Katz Sand I. (2017). The use of natalizumab for multiple sclerosis. Neuropsychiatr. Dis. Treat. 13, 1691–1702. 10.2147/NDT.S114636 28721050 PMC5499927

[B12] Camussi G. Deregibus M. C. Bruno S. Cantaluppi V. Biancone L. (2010). Exosomes/microvesicles as a mechanism of cell-to-cell communication. Kidney Int. 78, 838–848. 10.1038/ki.2010.278 20703216

[B13] Carandini T. Colombo F. Finardi A. Casella G. Garzetti L. Verderio C.. (2015). Microvesicles: what is the role in multiple sclerosis? Front. Neurol. 6:111. 10.3389/fneur.2015.00111 26074867 PMC4443736

[B14] Caruso S. Poon I. K. H. (2018). Apoptotic cell-derived extracellular vesicles: more than just debris. Front. Immunol. 9:1486. 10.3389/fimmu.2018.01486 30002658 PMC6031707

[B15] Casella G. Colombo F. Finardi A. Descamps H. Ill-Raga G. Spinelli A.. (2018). Extracellular vesicles containing IL-4 modulate neuroinflammation in a mouse model of multiple sclerosis. *Mol*. Ther. 26, 2107–2118. 10.1016/j.ymthe.2018.06.024 30017878 PMC6127510

[B16] Chargaff E. West R. (1946). The biological significance of the thromboplastic protein of blood. J. Biol. Chem. 166, 189–197. 20273687

[B17] Chiriacò M. S. Bianco M. Nigro A. Primiceri E. Ferrara F. Romano A. (2018). Lab-on-chip for exosomes and microvesicles detection and characterization. Sensors 18:3175 10.3390/s18103175 PMC6210978 30241303

[B18] Clark K. Zhang S. Barthe S. Kumar P. Pivetti C. Kreutzberg N.. (2019). Placental mesenchymal stem cell-derived extracellular vesicles promote myelin regeneration in an animal model of multiple sclerosis. Cells 8:1497. 10.3390/cells8121497 31771176 PMC6952942

[B19] Cocucci E. Meldolesi J. (2015). Ectosomes and exosomes: shedding the confusion between extracellular vesicles. Trends Cell Biol. 25, 364–372. 10.1016/j.tcb.2015.01.004 25683921

[B20] Cohen J. A. (2013). Mesenchymal stem cell transplantation in multiple sclerosis. *J. Neurol*. Sci. 333, 43–49. 10.1016/j.jns.2012.12.009 23294498 PMC3624046

[B21] Colombo M. Raposo G. Théry C. (2014). Biogenesis, secretion, and intercellular interactions of exosomes and other extracellular vesicles. Annu. Rev. Cell Dev. Biol. 30, 255–289. 10.1146/annurev-cellbio-101512-122326 25288114

[B22] Couch Y. Akbar N. Roodselaar J. Evans M. C. Gardiner C. Sargent I.. (2017). Circulating endothelial cell-derived extracellular vesicles mediate the acute phase response and sickness behaviour associated with CNS inflammation. Sci. Rep. 7, 1–12. 10.1038/s41598-017-09710-3 28851955 PMC5575066

[B23] Coumans F. A. W. Brisson A. R. Buzas E. I. Dignat-George F. Drees E. E. E. El-Andaloussi S.. (2017). Methodological guidelines to study extracellular vesicles. Circ. Res. 120, 1632–1648. 10.1161/CIRCRESAHA.117.309417 28495994

[B24] Crescitelli R. Lässer C. Jang S. C. Cvjetkovic A. Malmhäll C. Karimi N.. (2020). Subpopulations of extracellular vesicles from human metastatic melanoma tissue identified by quantitative proteomics after optimized isolation. *J. Extracell*. Vesicles 9:1722433. 10.1080/20013078.2020.1722433 32128073 PMC7034452

[B25] Crescitelli R. Lässer C. Szabó T. G. Kittel A. Eldh M. Dianzani I. (2013). Distinct RNA profiles in subpopulations of extracellular vesicles: apoptotic bodies, microvesicles and exosomes. J. Extracell. Vesicles 2. 10.3402/jev.v2i0.20677 PMC3823106 24223256

[B26] Dalla Costa G. Finardi A. Garzetti L. Carandini T. Comi G. Martinelli V.. (2018). Disease-modifying treatments modulate myeloid cells in multiple sclerosis patients. Neurol. Sci. Off. J. Ital. Neurol. Soc. Ital. Soc. Clin. Neurophysiol. 39, 373–376. 10.1007/s10072-017-3176-2 29185135

[B27] Dolcetti E. Bruno A. Guadalupi L. Rizzo F. R. Musella A. Gentile A.. (2020). Emerging role of extracellular vesicles in the pathophysiology of multiple sclerosis. Int. J. Mol. Sci. 21:7336. 10.3390/ijms21197336 33020408 PMC7582271

[B28] Dutta S. del Rosario I. Paul K. Palma J. A. Perlman S. L. Poon W. W. (2018). α-Synuclein in brain-derived blood exosomes distinguishes multiple system atrophy from Parkinson's disease. Ann. Neurol. 84, S191–S191.

[B29] Ebrahimkhani S. Beadnall H. N. Wang C. Suter C. M. Barnett M. H. Buckland M. E.. (2020). Serum exosome MicroRNAs predict multiple sclerosis disease activity after fingolimod treatment. Mol. Neurobiol. 57, 1245–1258. 10.1007/s12035-019-01792-6 31721043

[B30] Ebrahimkhani S. Vafaee F. Young P. E. Hur S. S. J. Hawke S. Devenney E.. (2017). Exosomal microRNA signatures in multiple sclerosis reflect disease status. *Sci*. Rep. 7, 1–10. 10.1038/s41598-017-14301-3 29084979 PMC5662562

[B31] Frühbeis C. Fröhlich D. Krämer-Albers E.-M. (2012). Emerging roles of exosomes in neuron–glia communication. Front. Physiol. 3:119. 10.3389/fphys.2012.00119 22557979 PMC3339323

[B32] Galazka G. Mycko M. Selmaj I. Raine C. Selmaj K. (2017). Multiple sclerosis: serum-derived exosomes express myelin proteins. Mult. Scler. J. 24:135245851769659. 10.1177/1352458517696597 28273783

[B33] García-Romero N. Carrión-Navarro J. Esteban-Rubio S. Lázaro-Ibáñez E. Peris-Celda M. Alonso M. M.. (2017). DNA sequences within glioma-derived extracellular vesicles can cross the intact blood-brain barrier and be detected in peripheral blood of patients. Oncotarget 8, 1416–1428. 10.18632/oncotarget.13635 27902458 PMC5352065

[B34] Gardiner C. Di Vizio D. Sahoo S. Théry C. Witwer K. W. Wauben M. (2016). Techniques used for the isolation and characterization of extracellular vesicles: results of a worldwide survey. *J. Extracell*. Vesicles 5:32945 10.3402/jev.v5.32945 PMC5090131 27802845

[B35] Gatson N. N. Williams J. L. Powell N. D. McClain M. A. Hennon T. R. Robbins P. D.. (2011). Induction of pregnancy during established EAE halts progression of CNS autoimmune injury via pregnancy-specific serum factors. J. Neuroimmunol. 230, 105–113. 10.1016/j.jneuroim.2010.09.010 20950868 PMC3021646

[B36] Geraci F. Ragonese P. Barreca M. M. Aliotta E. Mazzola M. A. Realmuto S.. (2018). Differences in intercellular communication during clinical relapse and gadolinium-enhanced MRI in patients with relapsing remitting multiple sclerosis: a study of the composition of extracellular vesicles in cerebrospinal fluid. Front. Cell. Neurosci. 12:418. 10.3389/fncel.2018.00418 30498433 PMC6249419

[B37] Giovannelli I. Martelli F. Repice A. Massacesi L. Azzi A. Giannecchini S. (2015). Detection of JCPyV microRNA in blood and urine samples of multiple sclerosis patients under natalizumab therapy. *J*. Neurovirol. 21, 666–670. 10.1007/s13365-015-0325-3 25678142

[B38] Goetzl E. J. Boxer A. Schwartz J. B. Abner E. L. Petersen R. C. Miller B. L.. (2015). Altered lysosomal proteins in neural-derived plasma exosomes in preclinical Alzheimer disease. Neurology 85, 40–47. 10.1212/WNL.0000000000001702 26062630 PMC4501943

[B39] Goetzl E. J. Elahi F. M. Mustapic M. Kapogiannis D. Pryhoda M. Gilmore A.. (2019). Altered levels of plasma neuron-derived exosomes and their cargo proteins characterize acute and chronic mild traumatic brain injury. FASEB J. 33, 5082–5088. 10.1096/fj.201802319R 30605353 PMC6436652

[B40] Goetzl E. J. Mustapic M. Kapogiannis D. Eitan E. Lobach I. V. Goetzl L.. (2016). Cargo proteins of plasma astrocyte-derived exosomes in Alzheimer's disease. FASEB J. 30, 3853–3859. 10.1096/fj.201600756R 27511944 PMC5067254

[B41] Goetzl E. J. Schwartz J. B. Abner E. L. Jicha G. A. Kapogiannis D. (2018). High complement levels in astrocyte-derived exosomes of Alzheimer disease. Ann. Neurol. 83, 544–552. 10.1002/ana.25172 29406582 PMC5867263

[B42] Harbo H. F. Gold R. Tintoré M. (2013). Sex and gender issues in multiple sclerosis. *Ther. Adv. Neurol*. Disord. 6, 237–248. 10.1177/1756285613488434 23858327 PMC3707353

[B43] Havrdova E. Galetta S. Hutchinson M. Stefoski D. Bates D. Polman C. H.. (2009). Effect of natalizumab on clinical and radiological disease activity in multiple sclerosis: a retrospective analysis of the Natalizumab Safety and Efficacy in Relapsing-Remitting Multiple Sclerosis (AFFIRM) study. Lancet Neurol. 8, 254–260. 10.1016/S1474-4422(09)70021-3 19201654

[B44] Hernandez-Oller L. Seras-Franzoso J. Andrade F. Rafael D. Abasolo I. Gener P.. (2020). Extracellular vesicles as drug delivery systems in cancer. Pharmaceutics 12:1146. 10.3390/pharmaceutics12121146 33256036 PMC7761384

[B45] Hornung S. Dutta S. Bitan G. (2020). CNS-derived blood exosomes as a promising source of biomarkers: opportunities and challenges. *Front. Mol*. Neurosci. 13:38. 10.3389/fnmol.2020.00038 32265650 PMC7096580

[B46] Jeppesen D. K. Fenix A. M. Franklin J. L. Higginbotham J. N. Zhang Q. Zimmerman L. J.. (2019). Reassessment of exosome composition. Cell 177, 428–445.e18. 10.1016/j.cell.2019.02.029 30951670 PMC6664447

[B47] Karimi N. Cvjetkovic A. Jang S. C. Crescitelli R. Hosseinpour Feizi M. A. Nieuwland R.. (2018). Detailed analysis of the plasma extracellular vesicle proteome after separation from lipoproteins. Cell. Mol. Life Sci. 75, 2873–2886. 10.1007/s00018-018-2773-4 29441425 PMC6021463

[B48] Karussis D. Karageorgiou C. Vaknin-Dembinsky A. Gowda-Kurkalli B. Gomori J. M. Kassis I.. (2010). Safety and immunological effects of mesenchymal stem cell transplantation in patients with multiple sclerosis and amyotrophic lateral sclerosis. *Arch*. Neurol. 67, 1187–1194. 10.1001/archneurol.2010.248 20937945 PMC3036569

[B49] Kibria G. Ramos E. Lee K. Bedoyan S. Huang S. Samaeekia R. (2016). A rapid, automated surface protein profiling of single circulating exosomes in human blood. Sci. Rep. 6:36502 10.1038/srep36502 27819324 PMC5098148

[B50] Kim H.-S. Choi D.-Y. Yun S. J. Choi S.-M. Kang J. W. Jung J. W.. (2012). Proteomic analysis of microvesicles derived from human mesenchymal stem cells. *J*. Proteome Res. 11, 839–849. 10.1021/pr200682z 22148876

[B51] Kim H. J. Park J.-S. (2017). Usage of human mesenchymal stem cells in cell-based therapy: advantages and disadvantages. Dev. Reprod. 21, 1–10. 10.12717/DR.2017.21.1.001 28484739 PMC5409204

[B52] Kimura K. Hohjoh H. Fukuoka M. Sato W. Oki S. Tomi C.. (2018). Circulating exosomes suppress the induction of regulatory T cells via let-7i in multiple sclerosis. Nat. Commun. 9:2406. 10.1038/s41467-017-02406-2 29295981 PMC5750223

[B53] Kowal J. Arras G. Colombo M. Jouve M. Morath J. P. Primdal-Bengtson B.. (2016). Proteomic comparison defines novel markers to characterize heterogeneous populations of extracellular vesicle subtypes. Proc. Natl. Acad. Sci. U.S.A. 113, E968–977. 10.1073/pnas.1521230113 26858453 PMC4776515

[B54] Lässer C. Jang S. C. Lötvall J. (2018). Subpopulations of extracellular vesicles and their therapeutic potential. Mol. Aspects Med. 60, 1–14. 10.1016/j.mam.2018.02.002 29432782

[B55] Lassmann H. (2019). Pathogenic mechanisms associated with different clinical courses of multiple sclerosis. Front. Immunol. 9:3116. 10.3389/fimmu.2018.03116 30687321 PMC6335289

[B56] Lebrun-Frenay C. Kantarci O. Siva A. Sormani M. P. Pelletier D. Okuda D. T.. (2020). Radiologically isolated syndrome: 10-year risk estimate of a clinical event. Ann. Neurol. 88, 407–417. 10.1002/ana.25799 32500558

[B57] Lee Y. X. F. Johansson H. Wood M. J. A. El Andaloussi S. (2019). Considerations and implications in the purification of extracellular vesicles – a cautionary tale. Front. Neurosci. 13:1067. 10.3389/fnins.2019.01067 31680809 PMC6813730

[B58] Li S. Xu J. Qian J. Gao X. (2020). Engineering extracellular vesicles for cancer therapy: recent advances and challenges in clinical translation. Biomater. Sci. 8, 6978–6991. 10.1039/d0bm01385d 33155579

[B59] Li X. Zhang Y. Yan Y. Ciric B. Ma C.-G. Chin J. (2017). LINGO-1-Fc-transduced neural stem cells are effective therapy for chronic stage experimental autoimmune encephalomyelitis. *Mol*. Neurobiol. 54, 4365–4378. 10.1007/s12035-016-9994-z 27344330

[B60] Li Z. Liu F. He X. Yang X. Shan F. Feng J. (2019). Exosomes derived from mesenchymal stem cells attenuate inflammation and demyelination of the central nervous system in EAE rats by regulating the polarization of microglia. Int. Immunopharmacol. 67, 268–280. 10.1016/j.intimp.2018.12.001 30572251

[B61] Lombardi M. Parolisi R. Scaroni F. Bonfanti E. Gualerzi A. Gabrielli M.. (2019). Detrimental and protective action of microglial extracellular vesicles on myelin lesions: astrocyte involvement in remyelination failure. Acta Neuropathol. 138, 987–1012. 10.1007/s00401-019-02049-1 31363836 PMC6851224

[B62] Lötvall J. Hill A. F. Hochberg F. Buzás E. I. Di Vizio D. Gardiner C.. (2014). Minimal experimental requirements for definition of extracellular vesicles and their functions: a position statement from the International Society for Extracellular Vesicles. J. Extracell. Vesicles 3:26913. 10.3402/jev.v3.26913 25536934 PMC4275645

[B63] Lublin F. D. Reingold S. C. Cohen J. A. Cutter G. R. Sørensen P. S. Thompson A. J.. (2014). Defining the clinical course of multiple sclerosis. Neurology 83, 278–286. 10.1212/WNL.0000000000000560 24871874 PMC4117366

[B64] Lunavat T. R. Cheng L. Kim D.-K. Bhadury J. Jang S. C. Lässer C.. (2015). Small RNA deep sequencing discriminates subsets of extracellular vesicles released by melanoma cells – evidence of unique microRNA cargos. RNA Biol. 12, 810–823. 10.1080/15476286.2015.1056975 26176991 PMC4615768

[B65] Manna I. Iaccino E. Dattilo V. Barone S. Vecchio E. Mimmi S.. (2018). Exosome-associated miRNA profile as a prognostic tool for therapy response monitoring in multiple sclerosis patients. FASEB J. 32, 4241–4246. 10.1096/fj.201701533R 29505299

[B66] Marcos-Ramiro B. Oliva Nacarino P. Serrano-Pertierra E. Blanco-Gelaz M. Á. Weksler B. B. Romero I. A.. (2014). Microparticles in multiple sclerosis and clinically isolated syndrome: effect on endothelial barrier function. BMC Neurosci. 15:110. 10.1186/1471-2202-15-110 25242463 PMC4261570

[B67] Mathivanan S. Simpson R. (2009). ExoCarta: a compendium of exosomal proteins and RNA. Proteomics 9, 4997–5000. 10.1002/pmic.200900351 19810033

[B68] Moyano A. L. Li G. Boullerne A. I. Feinstein D. L. Hartman E. Skias D.. (2016). Sulfatides in extracellular vesicles isolated from plasma of multiple sclerosis patients. J. Neurosci. Res. 94, 1579–1587. 10.1002/jnr.23899 27557608

[B69] Muhsin-Sharafaldine M.-R. McLellan A. D. (2018). Tumor-derived apoptotic vesicles: with death they do part. Front. Immunol. 9:957. 10.3389/fimmu.2018.00957 29780392 PMC5952256

[B70] Muralidharan-Chari V. Clancy J. Plou C. Romao M. Chavrier P. Raposo G.. (2009). ARF6-regulated shedding of tumor cell-derived plasma membrane microvesicles. Curr. Biol. 19, 1875–1885. 10.1016/j.cub.2009.09.059 19896381 PMC3150487

[B71] Muralidharan-Chari V. Clancy J. W. Sedgwick A. D'Souza-Schorey C. (2010). Microvesicles: mediators of extracellular communication during cancer progression. *J*. Cell Sci. 123, 1603–1611. 10.1242/jcs.064386 20445011 PMC2864708

[B72] Mustapic M. Eitan E. Werner J. K. Berkowitz S. T. Lazaropoulos M. P. Tran J. (2017). Plasma extracellular vesicles enriched for neuronal origin: a potential window into brain pathologic processes. Front. Neurosci. 11:278 10.3389/fnins.2017.00278 28588440 PMC5439289

[B73] Nigro A. Colombo F. Casella G. Finardi A. Verderio C. Furlan R. (2016). Myeloid extracellular vesicles: messengers from the demented brain. Front. Immunol. 7:17. 10.3389/fimmu.2016.00017 26858720 PMC4731486

[B74] Niwald M. Migdalska-Sek M. Brzeziańska-Lasota E. Miller E. (2017). Evaluation of selected microRNAs expression in remission phase of multiple sclerosis and their potential link to cognition, depression, and disability. J. Mol. Neurosci. 63, 275–282. 10.1007/s12031-017-0977-y 29043654

[B75] Nordberg M. Eaton E. Gonzalez Toledo E. Harris M. Chalamidas K. McGee J.. (2011). The effects of high dose interferon-β1a on plasma microparticles: correlation with MRI parameters. J. Neuroinflamm. 8:43. 10.1186/1742-2094-8-43 21554694 PMC3120694

[B76] Onódi Z. Pelyhe C. Terézia Nagy C. Brenner G. B. Almási L. Kittel Á.. (2018). Isolation of high-purity extracellular vesicles by the combination of iodixanol density gradient ultracentrifugation and bind-elute chromatography from blood plasma. Front. Physiol. 9:1479. 10.3389/fphys.2018.01479 30405435 PMC6206048

[B77] Paul D. Baena V. Ge S. Jiang X. Jellison E. R. Kiprono T.. (2016). Appearance of claudin-5+ leukocytes in the central nervous system during neuroinflammation: a novel role for endothelial-derived extracellular vesicles. J. Neuroinflamm. 13:292. 10.1186/s12974-016-0755-8 27852330 PMC5112695

[B78] Pieragostino D. Cicalini I. Lanuti P. Ercolino E. di Ioia M. Zucchelli M.. (2018). Enhanced release of acid sphingomyelinase-enriched exosomes generates a lipidomics signature in CSF of multiple sclerosis patients. Sci. Rep. 8:3071. 10.1038/s41598-018-21497-5 29449691 PMC5814401

[B79] Pieragostino D. Lanuti P. Cicalini I. Cufaro M. C. Ciccocioppo F. Ronci M.. (2019). Proteomics characterization of extracellular vesicles sorted by flow cytometry reveals a disease-specific molecular cross-talk from cerebrospinal fluid and tears in multiple sclerosis. J. Proteomics 204:103403. 10.1016/j.jprot.2019.103403 31170500

[B80] Prada I. Gabrielli M. Turola E. Iorio A. D'Arrigo G. Parolisi R.. (2018). Glia-to-neuron transfer of miRNAs via extracellular vesicles: a new mechanism underlying inflammation-induced synaptic alterations. Acta Neuropathol. 135, 529–550. 10.1007/s00401-017-1803-x 29302779 PMC5978931

[B81] Pusic A. D. Pusic K. M. Kraig R. P. (2014). What are exosomes and how can they be used in multiple sclerosis therapy? *Expert Rev*. Neurother. 14, 353–355. 10.1586/14737175.2014.890893 24552578 PMC4090348

[B82] Ransohoff R. M. Hafler D. A. Lucchinetti C. F. (2015). Multiple sclerosis-a quiet revolution. *Nat. Rev*. Neurol. 11, 134–142. 10.1038/nrneurol.2015.14 PMC4556342 25686758

[B83] Raposo G. Stoorvogel W. (2013). Extracellular vesicles: exosomes, microvesicles, and friends. J. Cell Biol. 200, 373–383. 10.1083/jcb.201211138 23420871 PMC3575529

[B84] Record M. Subra C. Silvente-Poirot S. Poirot M. (2011). Exosomes as intercellular signalosomes and pharmacological effectors. Biochem. Pharmacol. 81, 1171–1182. 10.1016/j.bcp.2011.02.011 21371441

[B85] Rekker K. Saare M. Roost A. M. Kubo A.-L. Zarovni N. Chiesi A.. (2014). Comparison of serum exosome isolation methods for microRNA profiling. Clin. Biochem. 47, 135–138. 10.1016/j.clinbiochem.2013.10.020 24183884

[B86] Riazifar M. Mohammadi M. R. Pone E. J. Yeri A. Lässer C. Segaliny A. I.. (2019). Stem cell-derived exosomes as nanotherapeutics for autoimmune and neurodegenerative disorders. ACS Nano 13, 6670–6688. 10.1021/acsnano.9b01004 31117376 PMC6880946

[B87] Sáenz-Cuesta M. Alberro A. Muñoz-Culla M. Osorio-Querejeta I. Fernandez-Mercado M. Lopetegui I.. (2018). The first dose of fingolimod affects circulating extracellular vesicles in multiple sclerosis patients. Int. J. Mol. Sci. 19:2448. 10.3390/ijms19082448 30126230 PMC6121302

[B88] Sáenz-Cuesta M. Osorio-Querejeta I. Otaegui D. (2014). Extracellular vesicles in multiple sclerosis: what are they telling us? Front. Cell. Neurosci. 8:100. 10.3389/fncel.2014.00100 24734004 PMC3975116

[B89] Scolding N. J. Morgan B. P. Houston W. A. Linington C. Campbell A. K. (1989). Vesicular removal by oligodendrocytes of membrane attack complexes formed by activated complement. Nature 339, 620–622. 10.1038/339620a0 2733792

[B90] Selmaj I. Mycko M. P. Raine C. S. Selmaj K. W. (2017). The role of exosomes in CNS inflammation and their involvement in multiple sclerosis. J. Neuroimmunol. 306, 1–10. 10.1016/j.jneuroim.2017.02.002 28385180

[B91] Shi M. Sheng L. Stewart T. Zabetian C. P. Zhang J. (2019). New windows into the brain: central nervous system-derived extracellular vesicles in blood. *Prog*. Neurobiol. 175, 96–106. 10.1016/j.pneurobio.2019.01.005 30685501 PMC6546433

[B92] Stuffers S. Brech A. Stenmark H. (2009). ESCRT proteins in physiology and disease. Exp. Cell Res. 315, 1619–1626. 10.1016/j.yexcr.2008.10.013 19013455

[B93] Szatanek R. Baj-Krzyworzeka M. Zimoch J. Lekka M. Siedlar M. Baran J. (2017). The methods of choice for extracellular vesicles (EVs) characterization. *Int. J. Mol*. Sci. 18:1153. 10.3390/ijms18061153 28555055 PMC5485977

[B94] Takov K. Yellon D. M. Davidson S. M. (2019). Comparison of small extracellular vesicles isolated from plasma by ultracentrifugation or size-exclusion chromatography: yield, purity and functional potential. J. Extracell. Vesicles 8:1560809 10.1080/20013078.2018.1560809 30651940 PMC6327926

[B95] Tang Y.-T. Huang Y.-Y. Zheng L. Qin S.-H. Xu X.-P. An T.-X.. (2017). Comparison of isolation methods of exosomes and exosomal RNA from cell culture medium and serum. *Int. J. Mol*. Med. 40, 834–844. 10.3892/ijmm.2017.3080 28737826 PMC5548045

[B96] Théry C. Amigorena S. Raposo G. Clayton A. (2006). Isolation and characterization of exosomes from cell culture supernatants and biological fluids. Isol. Charact. Exosomes Cell Cult. Supernatants Biol. 30, 3.22.1-3.22.29. 10.1002/0471143030.cb0322s30 18228490

[B97] Théry C. Witwer K. W. Aikawa E. Alcaraz M. J. Anderson J. D. Andriantsitohaina R.. (2018). Minimal information for studies of extracellular vesicles 2018 (MISEV2018): a position statement of the International Society for Extracellular Vesicles and update of the MISEV2014 guidelines. J. Extracell. Vesicles 7:1535750. 10.1080/20013078.2018.1535750 30637094 PMC6322352

[B98] Thompson A. G. Gray E. Heman-Ackah S. M. Mäger I. Talbot K. Andaloussi S. E.. (2016). Extracellular vesicles in neurodegenerative disease — pathogenesis to biomarkers. *Nat. Rev*. Neurol. 12, 346–357. 10.1038/nrneurol.2016.68 27174238

[B99] Tkach M. Théry C. (2016). Communication by extracellular vesicles: where we are and where we need to go. Cell 164, 1226–1232. 10.1016/j.cell.2016.01.043 26967288

[B100] Tricarico C. Clancy J. D'Souza-Schorey C. (2016). Biology and biogenesis of shed microvesicles. Small GTPases. 8, 220–232. 10.1080/21541248.2016.1215283 27494381 PMC5680703

[B101] van der Pol E. Sturk A. van Leeuwen T. Nieuwland R. Coumans F. ISTH-SSC-VB Working Group (2018). Standardization of extracellular vesicle measurements by flow cytometry through vesicle diameter approximation. J. Thromb. Haemost. 16, 1236–1245. 10.1111/jth.14009 29575716

[B102] van der Vos K. E. Abels E. R. Zhang X. Lai C. Carrizosa E. Oakley D.. (2016). Directly visualized glioblastoma-derived extracellular vesicles transfer RNA to microglia/macrophages in the brain. Neuro Oncol. 18, 58–69. 10.1093/neuonc/nov244 26433199 PMC4677420

[B103] van Niel G. D'Angelo G. Raposo G. (2018). Shedding light on the cell biology of extracellular vesicles. *Nat. Rev. Mol*. Cell Biol. 19, 213–228. 10.1038/nrm.2017.125 29339798

[B104] Verderio C. Muzio L. Turola E. Bergami A. Novellino L. Ruffini F.. (2012). Myeloid microvesicles are a marker and therapeutic target for neuroinflammation. Ann. Neurol. 72, 610–624. 10.1002/ana.23627 23109155

[B105] Villa F. Quarto R. Tasso R. (2019). Extracellular vesicles as natural, safe and efficient drug delivery systems. Pharmaceutics 11:557. 10.3390/pharmaceutics11110557 31661862 PMC6920944

[B106] Weiner H. L. (2008). A shift from adaptive to innate immunity: a potential mechanism of disease progression in multiple sclerosis. J. Neurol. 255(Suppl. 1), 3–11. 10.1007/s00415-008-1002-8 18317671

[B107] Welton J. L. Loveless S. Stone T. von Ruhland C. Robertson N. P. Clayton A. (2017). Cerebrospinal fluid extracellular vesicle enrichment for protein biomarker discovery in neurological disease; multiple sclerosis. J. Extracell. Vesicles 6:1369805. 10.1080/20013078.2017.1369805 28959386 PMC5614217

[B108] Wheway J. Latham S. L. Combes V. Grau G. E. R. (2014). Endothelial microparticles interact with and support the proliferation of T cells. J. Immunol. 193, 3378–3387. 10.4049/jimmunol.1303431 25187656 PMC4170003

[B109] Wickman G. Julian L. Olson M. F. (2012). How apoptotic cells aid in the removal of their own cold dead bodies. Cell Death Differ. 19, 735–742. 10.1038/cdd.2012.25 22421963 PMC3321633

[B110] Williams J. L. Gatson N. N. Smith K. M. Almad A. McTigue D. M. Whitacre C. C. (2013). Serum exosomes in pregnancy-associated immune modulation and neuroprotection during CNS autoimmunity. *Clin. Immunol*. Orlando Fla 149, 236–243. 10.1016/j.clim.2013.04.005 23706172 PMC3778091

[B111] Willms E. Cabañas C. Mäger I. Wood M. J. A. Vader P. (2018). Extracellular vesicle heterogeneity: subpopulations, isolation techniques, and diverse functions in cancer progression. Front. Immunol. 9:738. 10.3389/fimmu.2018.00738 29760691 PMC5936763

[B112] Xu X. Lai Y. Hua Z.-C. (2019). Apoptosis and apoptotic body: disease message and therapeutic target potentials. Biosci. Rep. 39:BSR20180992. 10.1042/BSR20180992 30530866 PMC6340950

[B113] Yamout B. Hourani R. Salti H. Barada W. El-Hajj T. Al-Kutoubi A.. (2010). Bone marrow mesenchymal stem cell transplantation in patients with multiple sclerosis: a pilot study. J. Neuroimmunol. 227, 185–189. 10.1016/j.jneuroim.2010.07.013 20728948

[B114] Yáñez,-Mó M. Siljander P. R.-M. Andreu Z. Zavec A. B. Borràs F. E. Buzas E. I.. (2015). Biological properties of extracellular vesicles and their physiological functions. *J. Extracell*. Vesicles 4:27066. 10.3402/jev.v4.27066 25979354 PMC4433489

[B115] Yu L. Yang F. Jiang L. Chen Y. Wang K. Xu F.. (2013). Exosomes with membrane-associated TGF-β1 from gene-modified dendritic cells inhibit murine EAE independently of MHC restriction. Eur. J. Immunol. 43, 2461–2472. 10.1002/eji.201243295 23716181

[B116] Zappulli V. Friis K. P. Fitzpatrick Z. Maguire C. A. Breakefield X. O. (2016). Extracellular vesicles and intercellular communication within the nervous system. J. Clin. Invest. 126, 1198–1207. 10.1172/JCI81134 27035811 PMC4811121

[B117] Zhang Q. Fu L. Liang Y. Guo Z. Wang L. Ma C.. (2018). Exosomes originating from MSCs stimulated with TGF-β and IFN-γ promote Treg differentiation. J. Cell Physiol. 233, 6832–6840. 10.1002/jcp.26436 29336475 PMC13482522

[B118] Zhang Y. Han J.-J. Liang X.-Y. Zhao L. Zhang F. Rasouli J.. (2018). miR-23b suppresses leukocyte migration and pathogenesis of experimental autoimmune encephalomyelitis by targeting CCL7. Mol. Ther. 26, 582–592. 10.1016/j.ymthe.2017.11.013 29275848 PMC5835026

[B119] Zhuang X. Xiang X. Grizzle W. Sun D. Zhang S. Axtell R. C.. (2011). Treatment of brain inflammatory diseases by delivering exosome encapsulated anti-inflammatory drugs from the nasal region to the brain. Mol. Ther. J. Am. Soc. Gene Ther. 19, 1769–1779. 10.1038/mt.2011.164 21915101 PMC3188748

[B120] Ziemssen T. Akgün K. Brück W. (2019). Molecular biomarkers in multiple sclerosis. J. Neuroinflamm. 16:272. 10.1186/s12974-019-1674-2 31870389 PMC6929340

[B121] Zinger A. Latham S. L. Combes V. Byrne S. Barnett M. H. Hawke S.. (2016). Plasma levels of endothelial and B-cell-derived microparticles are restored by fingolimod treatment in multiple sclerosis patients. Mult. Scler. J. 22, 1883–1887. 10.1177/1352458516636959 26931477

